# Significance of Kampo, Traditional Japanese Medicine, in Supportive Care of Cancer Patients

**DOI:** 10.1155/2013/746486

**Published:** 2013-06-19

**Authors:** Jun-ichi Yamakawa, Yoshiharu Motoo, Junji Moriya, Masao Ogawa, Hiroaki Uenishi, Sumiyo Akazawa, Toshiyuki Sasagawa, Matomo Nishio, Junji Kobayashi

**Affiliations:** ^1^Department of General Medicine, Kanazawa Medical University, 1-1 Daigaku, Uchinada, Ishikawa 920-0293, Japan; ^2^Department of Medical Oncology, Kanazawa Medical University, 1-1 Daigaku, Uchinada, Ishikawa 920-0293, Japan; ^3^Department of Anesthesiology, Kanazawa Medical University, 1-1 Daigaku, Uchinada, Ishikawa 920-0293, Japan; ^4^Department of Obstetrics and Gynecology, Kanazawa Medical University, 1-1 Daigaku, Uchinada, Ishikawa 920-0293, Japan; ^5^Department of Pharmacology, Kanazawa Medical University, 1-1 Daigaku, Uchinada, Ishikawa 920-0293, Japan

## Abstract

The current standard treatment for cancer is a multidisciplinary therapy whereby various types of treatment are properly combined. Chemotherapy with multiple anticancer drugs is now common, and traditional, complementary, and alternative therapies are adopted as supportive measures. Medical care in Japan is distinguished by the ability for patients to access both Western and Kampo medical cares at the same time. There is a high degree of trust in the safety of Kampo therapies because they are practiced by medical doctors who are educated with fundamental diagnosis of Western medicine. Highly reliable clinical studies are being published, demonstrating that palliative or supportive care for cancer patients using Kampo preparations alleviates adverse effects of chemotherapy or radiotherapy. This paper reports the circumstances around cancer care in Japan where traditional therapeutic Kampo formulas are used for patients undergoing cancer treatment with cutting-edge chemotherapy, specifically to alleviate adverse effects of anticancer drugs.

## 1. Background

### 1.1. Historical Background of Cancer Treatment in Japan

Surgery, radiotherapy, and chemotherapy are the main medical treatments for cancer. Chief among those is surgery. In recent years, advances have been made in a range of treatments that target specific characteristics and stages of cancer. By its nature, cancer develops after gene mutations in the body's cells, and the difficulty in treating cancer lies in the fact that cells metastasize. Surgery and radiation are local therapies, which leave the problem of how to treat the invisible remaining cancer cells. What is then required is not a localized treatment but a systemic treatment such as chemotherapy.

But until progress was made in the development of anticancer drugs, there was no effective treatment against cancer once it had spread throughout the body. A combination of surgery with chemotherapy is generally used. And sometimes radiotherapy is also used. Nowadays, the standard treatment is “multidisciplinary treatment [1–5],” a comprehensive form of treatment that efficiently combines a variety of treatments. 

### 1.2. Anticancer Drugs

Chemotherapy now occupies an important position in the treatment of cancer. Anticancer drugs have greatly changed cancer treatment. Excellent therapeutic effects have recently been achieved by combining radiation with anticancer drugs, even for solid cancers. The Achilles heel of anticancer drugs has been the strength of the adverse reactions [6–18]; however, these have been alleviated with the development of administration methods and supportive care to control nausea, vomiting, and so forth; therefore, patients do not suffer as much as before. Yet, the history of chemotherapy is still short. Surgery has been available for about 100 years and radiotherapy for about 50, but anticancer drugs have only been used to treat cancer for the last 35 years. 

Anticancer drugs have completely different effects depending on the type of cancer. While chemotherapy may be effective for some cancers, it is virtually ineffective for others. The effects of anticancer drugs also differ according to the way they are used. Potent effects are demonstrated when using drugs in combination, even if each anticancer drug does not promise sufficient effect when used alone. Nowadays, two to four types of anticancer drugs are used in combination to enhance their effectiveness, even at a modest amount. Such multidrug therapy is now being widely used and offers the hope of synergistic or additive effects.

### 1.3. Historical Background of Kampo Medicine

Traditional, complementary, and alternative therapies [19–25] are widely used and researched in the USA. Underlying this is the high cost of health care in that country and the common use of cheap folk remedies as well as traditional therapies and supplements against illness. The same situation exists in Europe and is becoming more widespread in Asia, where governments are promoting integrative medicine. There is a universal health insurance system which enables everybody in Japan to receive advanced health care at low cost. Therefore, alternative medicine did not attract attention. Japan's universal health insurance system [26, 27] is held in high regard across the country, and it means patients receive standard care for cancer at any medical service provider under this insurance system. However, if you prefer complementary or alternative therapies, you must pay a private provider out of your own pocket. 

Yet, another characteristic of medical care in Japan is that patients can access Western and Kampo medical cares at the same time. Kampo medicine [28–30] is a unique medical system that originated from ancient China, was gradually imported to Japan since approximately 1500 years ago, and has been improved and refined by many excellent physicians especially since the 17th century (Edo periods in Japanese era). Now, most Kampo preparations (Japanese traditional herbal medicines) are available as extract formulations of high quality, which are greatly different from herbal medicines used in China, Taiwan, and Korea, where most preparations are herbal decoctions. 

Four ethical Kampo extract formulations were approved in 1967 in Japan. Since then, the number of ethical Kampo extract formulations covered by health insurance has grown to 148. Much Japanese herbal extract preparation is used in Japan. Kampo extract preparation is mostly used in Japan. These Kampo extracts are the combination of herbal medicines from the viewpoint of Kampo theory. Standard examinations are done, and quality control of index ingredient is displayed. Kampo formulation for prescription is used for cancer medical treatment. Japan's universal health insurance system does allow simultaneous access to traditional Kampo preparations and Western medicines. However, doctors in Japan cannot be licensed without passing a board examination of Western medicine, which means patients in this country receive health care with a high degree of safety. This is another factor that distinguishes the health care system in Japan from other countries. In Japan, physicians who have studied Western medicine and Kampo medicine practice these approaches in their medical treatment of cancer with the aim of fusing Eastern and Western medicines into a unitary medical system, unlike the dual medical systems in China or Korea.

### 1.4. Supportive Care for Cancer Patients Using Kampo Preparations

Some people involved in the treatment of cancer reject Kampo therapy. The biggest reason they give is the scarcity of evidence. Kampo medicine is fundamentally a tailor-made type of treatment, and Kampo prescription is changed according to the patients' condition and symptoms. Therefore, the benefits of Kampo preparations cannot be fully evaluated using the criteria of the randomized clinical trials as in Western medicine. Objective data and proof of action mechanisms are required. Most of the studies on the actions of Kampo preparations have been animal trials and small-scale clinical trials. Little research has been done that offers highly reliable evidence, although progress has been made in this area recently [31–37]. The use of Kampo preparations for palliative and supportive care of cancer patients in combination with anticancer drugs or radiotherapy may offer alleviation of adverse effects and survival benefits, and the number of such research papers being published in international journals is increasing. 

## 2. Kampo for Chemotherapy-Induced Peripheral Neuropathy

### 2.1. Cancer Chemotherapy-Induced Peripheral Neuropathy

A drawback of most anticancer drugs currently in use is that they are not cancer cell-specific: their actions affect all multiplying cells. They interfere with cancer cell division by interfering with DNA replication and the functioning of the proteins necessary for cell division, but they also damage normal cells. Myeloid cells, immune cells, gastrointestinal mucosal cells, and hair root cells are particularly susceptible to damage and are prone to adverse effects such as bone marrow suppression, immunodeficiency, digestive symptoms, and alopecia. Since nerve and muscle cells do not undergo cell division, they are thought to be robust against such damage. However, some anticancer drugs are known to cause peripheral neuropathy. While it is only certain anticancer drugs that has this side effect, we know that patients who take the following drugs develop peripheral neuropathy: taxane-based drugs [38–43] such as paclitaxel and docetaxel; vinca alkaloids such as vincristine sulfate; and platinum-based drugs [41, 44–55] including cisplatin and oxaliplatin. The causes involve injury to axonal microtubules and direct injury to nerve cells. Microtubules are necessary for the transfer of chromosomes when cells divide. If the formation of microtubules is disturbed, cell division is inhibited. In addition, microtubules are also found within axons, which transmit nerve cell signals, and are involved in axonal development and material transportation. Vinca alkaloids and taxanes, in particular, act on the microtubules within cancer cells but cause neuropathy because they simultaneously damage the microtubules in normal nerve cells. Platinum-based drugs directly damage nerve cells and are thought to lead to nerve cell axon disorder.

### 2.2. Medical Treatment of Peripheral Neuropathy

Peripheral neuropathy symptoms include limb extremity numbness, as well as sensory motor ataxia, deep tendon reflex decline, and decreased muscle strength. There is great variation among sufferers of such complaints because sensation of these symptoms is extremely subjective. Patients may variously feel a tingling or stinging numbness or pain in the toes or fingers; an electric, shooting pain; loss of sense of touch; loss of heat/cold sensation; loss of power in the arms/legs; difficulty in grasping objects; or they may fall when walking. There are few effective remedies once peripheral neuropathy appears as a result of chemotherapy. In some cases the neuropathy may be almost irreversible. If symptoms are severe, the anticancer drug treatment must be discontinued or the prescription should be changed. In most cases, neuropathy persists as long as chemotherapy continues, and the symptoms do not disappear completely even after treatment ends, and complete recovery may take a long time. Treatment for peripheral neuropathy is not yet well established. Common medications including the combined use of calcium and magnesium or vitamin B6 and B12 have been reported to be effective to relieve numbness. The main symptomatic treatments for neuropathic pain include antidepressants, NSAIDs, or serotonin and norepinephrine reuptake inhibitors. If pain is severe, morphine and other narcotic analgesics may also be prescribed [56]. 

### 2.3. Indications and Evidence for Kampo Therapy for Chemotherapy-Induced Peripheral Neuropathy

The use of the Kampo preparation goshajinkigan [57–67] as drug therapy for peripheral neuropathy symptoms has been widely reported in Japan. Goshajinkigan extract preparation has been reported to relieve symptoms such as numbness or pain in 80% of cases in which it is used for peripheral neuropathy caused by paclitaxel for breast cancer [68]. Goshajinkigan also improves subjective symptoms of peripheral neuropathy due to the combined use of paclitaxel and carboplatin for ovarian or uterine cancers. Neuropathy is a characteristic adverse effect of oxaliplatin, the core drug for colorectal cancer. A high incidence of symptoms such as extremity numbness and cold sensation has been observed with the continued therapeutic use of oxaliplatin, especially at a cumulative dose over 500 mg/m^2^. Treatment can be continued if symptoms are mild, but the dosage is decreased or the administration is discontinued in some severe cases. On the other hand, research has found that goshajinkigan can alleviate such symptoms. Nishioka et al. [69] and Kono et al. [70] conducted a retrospective comparison and examination of the effects of goshajinkigan for peripheral neuropathy associated with oxaliplatin in advanced or recurrent colorectal cancer patients. They found that the group which was administered goshajinkigan from the start of chemotherapy tolerated the largest dosage until onset of peripheral neuropathy. Goshajinkigan's efficacy differs according to the causal anticancer drug. It promises some effectiveness for numbness caused by paclitaxel, and so forth, but it is virtually ineffective for oxaliplatin. Since it might be effective for prevention of oxaliplatin-induced neuropathy, it would be better to administer goshajinkigan from the start of chemotherapy. It has been reported that administration of Kampo preparations promises an increase in the frequency of administration during the FOLFOX regimen, which centers on oxaliplatin, before onset of numbness as an adverse effect [58, 70]. 

### 2.4. Goshajinkigan

**Figure 1:**
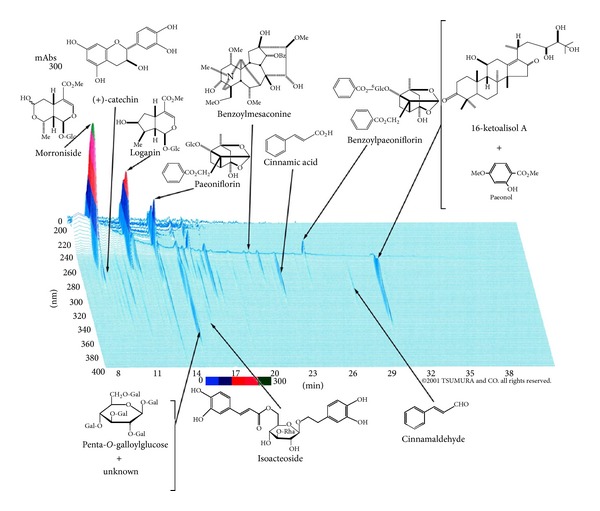
3D-HPLC pattern of TJ-107 goshajinkigan (this 3D-HPLC was created in 2001 by TSUMURA and CO.).

Goshajinkigan's Kampo constituents and HPLC fingerprint appear in Table 1 and Figure 1. 

Goshajinkigan is indicated for the relief of the following symptoms in patients with decreased urine volume or polyuria, occasional dry mouth, proneness to fatigue, and sensitivity to cold in the extremities: leg pain, low back pain, numbness, blurred vision (elderly), pruritus, dysuria, and edema. Goshajinkigan consists of 10 constituent crude drugs (Table 1) and is a prescription with fortified effectiveness against swelling, numbness, and arthralgia, in addition to the beneficial effects of hachimijiogan. Specifically, goshajinkigan is a Kampo preparation that improves blood circulation, has a body warming analgesic action, and reduces swelling. It is used for patients with remarkable edema tendency, severe arthralgia, and persistent low back pain. It is frequently used for symptoms in which peripheral vascular disease is suspected of being involved, such as sciatica and diabetic neuropathy, and has demonstrated effectiveness for these conditions. The usefulness of goshajinkigan is conjectured to be aconitine [71]. Shakuyakukanzoto is a Kampo preparation used for various types of myalgia including menstrual pain and cramp [72]. Shakuyakukanzoto has been reported to demonstrate effectiveness for arthralgia and myalgia due to paclitaxel [73]. While it is impossible to completely control peripheral neuropathy and myalgia caused by anticancer drugs, the combined use of goshajinkigan and shakuyakukanzoto may enhance improvement of subjective symptoms.

## 3. Kampo for Chemotherapy-Induced Diarrhea

### 3.1. Development of a Camptothecin Derivative—Irinotecan

Irinotecan is an anticancer drug classified as a plant alkaloid. It inhibits cancer cell proliferation by breaking DNA during cell division through its inhibition of the enzyme topoisomerase, which is required when DNA replicates. Wall et al. extracted and isolated camptothecin (CPT) in 1966 from *Camptotheca acuminate*, a plant native to China, and found that it has a powerful antineoplastic effect [74]. Subsequent development was undertaken by the National Cancer Institute (NCI) in the USA but was abandoned following the emergence of adverse effects. Pharmaceutical manufacturers in Japan vigorously pursued synthetic research into derivatives to preserve CPT's activity while reducing its toxicity, resulting in the CPT derivative irinotecan, which has been subsequently used as a potent anticancer drug. Irinotecan has demonstrated usefulness for various types of cancer, including colon and lung cancers, and its applications have been widening. Irinotecan suppresses the action of topoisomerase 1, which is involved in DNA replication, thereby demonstrating a strong antitumor effect; however, it can cause severe adverse effects including leukopenia and diarrhea [16, 17, 75–94]. 

### 3.2. Adverse Effects of Irinotecan and Their Frequency

The chief adverse effects are severe myelosuppression and intractable diarrhea. There have been reports of death following severe infection due to myelosuppression, intractable diarrhea, and intestinal perforation due to intestinal paralysis or bowel obstruction. Irinotecan undergoes metabolism in the liver where it is converted into the active metabolite SN-38, setting off an antitumor action. SN-38 is then deactivated by conjugation reaction by uridine diphosphate glucuronosyltransferase (UGT) and excreted into the duodenum through the biliary tract. However, individual variability in the UGT activity is thought to be a reason for individual variation in the adverse effects of irinotecan. Many reports [95–99] in recent years mention the relation between UGT1A1 genetic polymorphism and onset of adverse effects of irinotecan. UGT1A1 is a molecular species of UGT in the liver and is the enzyme that metabolizes irinotecan. UGT1A1*28 and *6 are variants of UGT1A1, and reports cite an increase in the incidence of severe adverse effects of irinotecan due to reduced UGT1A1 activity. 

The most troublesome adverse effect of CPT-11 is severe delayed diarrhea, which is caused by reactivation in the digestive tract by enteric bacterial *β*-glucuronidase. CPT-11 is a prodrug firstly decomposed by carboxyl esterase in the liver into SN-38, which has a powerful anticancer action and is then transported throughout the body. The SN-38 formed in the liver is glucuronidated by a glucuronidation enzyme also present in the liver. At this point, the SN-38 is deactivated, losing its injurious effect. However, after being excreted into the digestive tract via the biliary tract, the SN-38 is decomposed by enteric bacterial *β*-glucuronidase, thereby reforming SN-38. It is surmised that this SN-38 formed in the digestive tract then damages intestinal mucosal epithelial cells, giving rise to delayed diarrhea. 

### 3.3. Irinotecan Hydrochloride-Induced Diarrhea and Kampo

The flavonoid glycoside baicalin may control irinotecan hydrochloride-induced diarrhea because it actively inhibits *β*-glucuronidase of intestinal flora and suppresses reformation of the active form (SN-38) in the digestive tract. Large amounts of baicalin are contained in *Scutellaria* root, a constituent crude drug of Kampo preparations. Researchers have tested hangeshashinto for diarrhea as it is a Kampo preparation containing *Scutellaria* root. It has been reported in human clinical trials and animal experiments that administration of hangeshashinto [37, 100–104] extract formulation two to three days before irinotecan hydrochloride administration effectively prevents or reduces diarrhea. It has been ascertained that this does not affect the antitumor action. 

If the preventative effect against irinotecan hydrochloride-induced diarrhea is contained solely in the action mechanism of the flavonoid glycoside-induced *β*-glucuronidase inhibition, single administration of a flavonoid glycoside or *Scutellaria* root, rather than a Kampo formulation, may also be effective. Nevertheless, the comprehensive actions of the other crude drugs contained in hangeshashinto improve effectiveness. Specifically, it has been reported that hangeshashinto suppresses elevation of enteric prostaglandin E2, promotes repair of damaged intestinal mucosa, and improves intestinal water absorption [37, 100]. A particular characteristic of Kampo preparations is that they give greater efficacy through the synergistic effect of multiple crude drugs compared to one constituent alone. Loperamide hydrochloride is often administered for irinotecan hydrochloride-induced diarrhea, yet in some cases it is ineffective, maybe because loperamide does not cure intestinal mucosal damage. The Tochigi Cancer Center Research Group published the results of a clinical study that compared the degree of diarrhea in 41 patients with advanced non-small-cell lung cancer following anticancer drug treatment with irinotecan hydrochloride and cisplatin. Eighteen patients were administered hangeshashinto while the control group (without hangeshashinto administration) consisted of 23 patients. No significant difference in diarrhea frequency or interval was observed between the hangeshashinto group and the nonadministration group; however, the frequency of severe grade three and four diarrheas was lower in the hangeshashinto group [37].

### 3.4. Hangeshashinto

**Figure 2:**
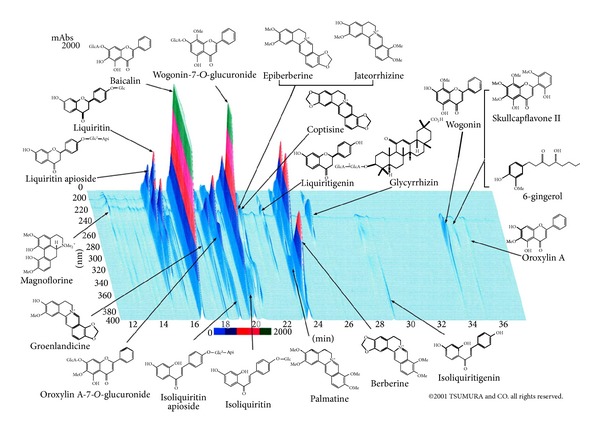
3D-HPLC pattern of TJ-14 hangeshashinto (this 3D-HPLC was created in 2001 by TSUMURA and CO.).

The Kampo constituents and HPLC fingerprint appear in Table 2 and Figure 2. 

Hangeshashinto is indicated for the relief of the following symptoms in those patients with blocked feeling in the stomach pit and occasional nausea, vomiting, anorexia, borborygmus, and a tendency of loose stool or diarrhea. The targeted diseases and symptoms are as follows: acute or chronic gastrointestinal catarrh, fermentative diarrhea, dyspepsia, gastroptosis, nervous gastritis, gastrasthenia, hangover, belching, heartburn, stomatitis, and neurosis. 

The reference sources for hangeshashinto are “*Shokanron*” and “*Kinki-yoryaku*”. Hangeshashinto consists of seven crude drugs. *Pinellia* Tuber clears fluid retention in the stomach and stops vomiting, while together with *Coptis* rhizome and *Scutellaria* root, it clears gastrointestinal inflammation. *Coptis* rhizome and *Scutellaria* root are bitter, and they are good for the stomach and have anti-inflammatory actions. *Ginseng* and ginger improve gastrointestinal blood flow and promote the recovery of gastrointestinal function. *Glycyrrhiza* and jujube harmonize crude drugs and enhance their cooperative effects.

## 4. Conclusions

Steady progress is being made in the treatment of cancer. However, highly invasive treatment can cause patients' distress. It would seem to be a natural progress that traditional, complementary, and alternative forms of medical care are now being adopted to alleviate the attendant suffering. Some express opposition to the combined use of Kampo preparations with anticancer drugs or surgery. Yet, if appropriate Kampo preparations alleviate adverse effects of cancer treatment, improve QOL, enhance therapeutic efficacy, and prolong life, the importance of treatment that includes Kampo preparations as palliative or supportive care for cancer will go on growing. Western and Kampo medicines coexist in Japan as a characteristic form of medical care. This combination needs to be further promoted and also to be established as integrative medicine based on scientific evidence.

## Figures and Tables

**Table 1:** Goshajinkigan extract granules for ethical use.

Description	Goshajinkigan extract granules for ethical use	
Composition	7.5 g of TSUMURA goshajinkigan extract granules contains 4.5 g of a dried extract of the following mixed crude drugs:	
	JP *Rehmannia* root	3.0 g
	JP *Achyranthes* root	3.0 g
	JP *Cornus* fruit	3.0 g
	JP *Dioscorea* rhizome	3.0 g
	JP *Plantago* seed	3.0 g
	JP *Alisma* rhizome	3.0 g
	JP poria sclerotium	3.0 g
	JP moutan bark	1.0 g
	JP cinnamon bark	1.0 g
	JP powdered processed aconite root	5.0 g
	Inactive ingredients	
	JP magnesium stearate	
	JP lactose hydrate	
	Sucrose esters of fatty acids	

(JP: The Japanese Pharmacopoeia.)

**Table 2:** Hangeshashinto extract granules for ethical use.

Description	Hangeshashinto extract granules for ethical use	
Composition	7.5 g of TSUMURA hangeshashinto extract granules contains 4.5 g of a dried extract of the following mixed crude drugs:	
	JP *Pinellia* tuber	5.0 g
	JP *Scutellaria* root	2.5 g
	JP processed ginger	2.5 g
	JP *Glycyrrhiza *	2.5 g
	JP jujube	2.5 g
	JP *Ginseng *	2.5 g
	JP *Coptis* rhizome	1.0 g
	Inactive ingredients	
	JP magnesium stearate	
	JP lactose hydrate	
	Sucrose esters of fatty acids	

(JP: The Japanese Pharmacopoeia.)
